# Atypical Pneumonia in Three Members of an Extended Family — South Carolina and North Carolina, July−August 2013

**Published:** 2014-08-22

**Authors:** Sarah K. Rhea, Stephanie W. Cox, Zack S. Moore, Ellen R. Mays, Alvaro J. Benitez, Maureen H. Diaz, Jonas M. Winchell

**Affiliations:** 1 Epidemic Intelligence Service, CDC; 2 North Carolina Department of Health and Human Services; 3 South Carolina Department of Health and Environmental Control; 4 Division of Bacterial Diseases, National Center for Immunizations and Respiratory Diseases, CDC

On August 5, 2013, the South Carolina Department of Health and Environmental Control was notified of a case of acute respiratory failure in a previously healthy woman. A family interview revealed the patient’s uncle and cousin had also been hospitalized with similar symptoms in North Carolina. The South Carolina Department of Health and Environmental Control and the North Carolina Division of Public Health collaborated to identify the cause of the respiratory illness cluster and to prevent additional illnesses.

The index patient (patient 1) was a woman aged 19 years and a resident of South Carolina. She was a smoker with no known prior medical conditions. During late July 2013, she experienced fever, shortness of breath, cough, and diarrhea and was hospitalized after 2 days of worsening respiratory symptoms. Chest radiographs from admission revealed diffuse bilateral infiltrates. Total white blood cell count was 25,000 with a neutrophil predominance. She subsequently experienced respiratory failure and required mechanical ventilation for 13 days. She received antibiotic treatment, including levofloxacin, azithromycin, cefepime, and vancomycin, and was discharged after a 17-day hospitalization. She recovered fully. No etiology was identified by laboratory testing, including bacterial cultures of blood and respiratory specimens, *Legionella* urinary antigen assay, and multiplex polymerase chain reaction (PCR) testing for influenza A and B, parainfluenza virus (PIV) 1–4, rhinovirus, adenovirus, human metapneumovirus, and respiratory syncytial virus (RSV).

Family interviews revealed that an uncle and cousin, both North Carolina residents, had experienced similar symptoms during the weeks before and after the index patient’s illness onset. Patient 2, aged 55 years, was the index patient’s uncle. He was a long-distance truck driver with a history of diabetes and obesity. He had experienced shortness of breath, cough, and fever in late June 2013, approximately 1 month before the index patient’s illness onset. After 5 days of worsening respiratory symptoms, he was hospitalized with bilateral pneumonia and progressive respiratory failure, for which he required mechanical ventilation. *Legionella* urinary antigen and bacterial cultures of respiratory specimens were negative. Chest radiographs revealed bilateral infiltrates. He received a single dose of ceftriaxone 4 days before hospitalization, and levofloxacin and piperacillin/tazobactam during a 14-day hospitalization. He recovered fully.

Patient 3, a cousin of the index patient and daughter of patient 2, was aged 26 years and had multiple risk factors for respiratory illness, including asthma, smoking, and pregnancy (33 weeks). She visited her father frequently during his hospitalization in late June. Four days before the index patient’s illness onset, patient 3 and the index patient traveled together by car for approximately 1 hour to attend the funeral of another family member who had died of unrelated causes. Two days after the index patient’s illness onset, patient 3 experienced shortness of breath, wheezing, and cough. Four days after these symptoms developed, patient 3 was hospitalized for progressive respiratory distress and placed on mechanical ventilation for respiratory failure. Chest radiographs revealed diffuse bilateral opacities. *Legionella* urinary antigen, bacterial cultures of respiratory specimens, and molecular testing for adenovirus, influenza types A and B, PIV1-3, and RSV were all negative. Patient 3 was prescribed azithromycin 1 day before hospitalization and received azithromycin and ceftriaxone during an 11-day hospitalization. She ultimately required mechanical ventilation for 6 days before making a complete recovery.

Patient 3’s infant was delivered prematurely by emergency Cesarean section at the time of her hospital admission. Upon delivery, the infant received a diagnosis of respiratory distress syndrome and possible sepsis; however, the complete blood count and C-reactive protein were not indicative of a bacterial infection. A blood culture was not performed. Ampicillin and gentamicin were administered during the infant’s 18-day hospitalization. He recovered fully.

As part of the public health investigation, upper airway aspirates and nasopharyngeal swabs from patients 1 and 3 were collected and submitted to CDC’s Pneumonia Response and Surveillance Laboratory for additional testing; respiratory specimens from patient 2 were unavailable. All specimens were tested by using a multiplex real-time PCR assay for simultaneous detection of *Mycoplasma pneumoniae*, *Chlamydophila pneumoniae*, *Legionella* species, and human nucleic acid control. *M. pneumoniae* was identified in both oropharyngeal and nasopharyngeal aspirates collected from patient 3 at 2 days and 8 days after her symptom onset. High-resolution melt analysis was used to determine susceptibility of the *M. pneumoniae* strain to macrolides on the basis of detection of a single nucleotide polymorphism within the 23S rRNA locus that confers resistance to this class of antibiotics. A profile consistent with macrolide sensitivity was observed in the *M. pneumoniae*–positive specimen. Further characterization by using multiple locus variable number tandem repeat analysis revealed a commonly observed strain type (3/5/6/2) in this patient. No respiratory pathogens were identified in specimens obtained from patient 1, which were collected 10 days after her symptom onset.

Although *M. pneumoniae* was only identified in clinical specimens from one patient in this cluster, the epidemiologic and clinical information collected during this investigation indicates that the organism was the likely cause of this cluster of atypical pneumonia. Pneumonia caused by *M. pneumoniae* typically has an incubation period of 1–3 weeks (1). All three patients had bilateral pulmonary infiltrates, lack of positive laboratory tests for other etiologies, and multiple opportunities for person-to-person spread within the family network. Two additional members of the extended family also experienced mild upper respiratory symptoms, including rhinorrhea and cough, in late July and early August 2013; however, neither sought medical care, and laboratory testing was not performed.

*M. pneumoniae* is a frequent cause of community-acquired pneumonia, and outbreaks of mild-to-moderate disease are common (2,3). Extrapulmonary manifestations of *M. pneumoniae* infection can contribute to severe disease and death (4). This disease cluster is remarkable because of the severity of illness, including the requirement for mechanical ventilation for all three patients. Risk factors for severe *M. pneumoniae* disease are not well-defined. However, conditions that compromise cardiopulmonary function (e.g., conditions present among the patients described) likely contributed (5–7). Testing for atypical bacterial respiratory pathogens (e.g., *M. pneumoniae*) should be considered when investigating clusters of community-acquired pneumonia, including clusters of severe disease. Increased awareness and availability of diagnostic tests at state and local public health laboratories might lead to improved understanding of the actual burden of this pathogen in the United States and its contributory role in outbreaks of severe respiratory illness.
